# Young infants with symptomatic tetralogy of Fallot: Shunt or primary repair?

**DOI:** 10.1016/j.xjon.2024.04.003

**Published:** 2024-04-11

**Authors:** Xin Tao Ye, Soichiro Henmi, Edward Buratto, Mitchell C. Haverty, Can Yerebakan, Tyson Fricke, Christian P. Brizard, Yves d’Udekem, Igor E. Konstantinov

**Affiliations:** a Department of Cardiac Surgery, Royal Children’s Hospital, Melbourne, Victoria, Australia; b Department of Paediatrics, University of Melbourne, Melbourne, Victoria, Australia; c Heart Research Group, Murdoch Children’s Research Institute, Melbourne, Victoria, Australia; d Division of Cardiac Surgery, Children's National Hospital, Washington, DC; e The George Washington University School of Medicine and Health Sciences, Washington, DC; f The Melbourne Centre for Cardiovascular Genomics and Regenerative Medicine, Murdoch Children’s Research Institute, Melbourne, Victoria, Australia

**Keywords:** tetralogy of Fallot, surgery, symptomatic, infants, palliation

## Abstract

**Objectives:**

The optimal treatment strategy for symptomatic young infants with tetralogy of Fallot (TOF) is unclear. We sought to compare the outcomes of staged repair (SR) (shunt palliation followed by second-stage complete repair) versus primary repair (PR) at 2 institutions that have exclusively adopted each strategy.

**Methods:**

We performed propensity score-matched comparison of 143 infants under 4 months of age who underwent shunt palliation at one institution between 1993 and 2021 with 122 infants who underwent PR between 2004 and 2018 at another institution. The primary outcome was mortality. Secondary outcomes were postoperative complications, durations of perioperative support and hospital stays, and reinterventions. Median follow-up was 8.3 years (interquartile range, 8.1-13.4 years).

**Results:**

After the initial procedure, hospital mortality (shunt, 2.8% vs PR, 2.5%; *P* = .86) and 10-year survival (shunt, 95%; 95% confidence interval [CI], 90%-98% vs PR, 90%; 95% CI, 81%-95%; *P* = .65) were similar. The SR group had a greater risk of early reinterventions but similar rates of late reinterventions. Propensity score matching yielded 57 well-balanced pairs. In the matched cohort, the SR group had similar freedom from reintervention (55%; 95% CI, 39%-68% vs 59%; 95% CI, 43%-71%; *P* = .85) and greater survival (98%; 95% CI, 88%-99.8% vs 85%; 95% CI, 69%-93%; *P* = .02) at 10 years, as the result of more noncardiac-related mortalities in the PR group.

**Conclusions:**

In symptomatic young infants with TOF operated at 2 institutions with exclusive treatment protocols, the SR strategy was associated with similar cardiac-related mortality and reinterventions as the PR strategy at medium-term follow-up.

**Figure:**
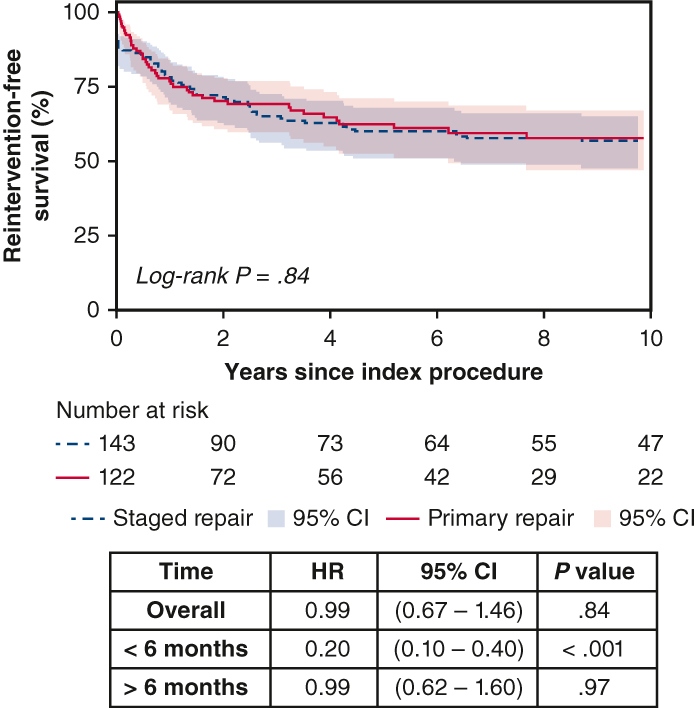
Reintervention-free survival after shunt palliation versus primary repair for symptomatic TOF.

**Central Message**

Cardiac-related mortality and late reinterventions do not differ between symptomatic young infants under 4 months of age undergoing staged repair versus primary repair for tetralogy of Fallot.

**Perspective**

The optimal treatment strategy for symptomatic infants with tetralogy of Fallot under 4 months of age is unclear, with some centers opting for early primary repair and others preferring staged repair preceded by shunt palliation. Our findings suggest that cardiac-related mortality and late reinterventions do not differ between the 2 strategies. Early reinterventions are more common after shunt palliation.

The optimal management strategy for symptomatic young infants with tetralogy of Fallot (TOF) remains controversial.^1^ Options of early intervention include staged repair (SR) or primary repair (PR). SR involves initial surgical or transcatheter palliation aimed at augmenting pulmonary blood flow followed by second-stage complete repair (CR). Proponents of PR have cited theoretical benefits of an earlier establishment of a normal biventricular circulation in optimizing pulmonary vasculature growth and brain development, arguing that early repair can be performed safely and effectively with comparable or reduced resource usage as staged palliation.^2^, ^3^, ^4^, ^5^, ^6^ Advocates of SR have argued that early repair is associated with excessive early morbidity and mortality and that initial palliation facilitates somatic growth of the pulmonary valve annulus and thereby reduces the need for a transannular incision at the time of repair.^1^^,^^4^^,^^7^, ^8^, ^9^, ^10^, ^11^ Others have proposed that the optimal strategy for each patient should be individualized according to the initial size of the pulmonary valve annulus and expertise of the institution.^12^, ^13^, ^14^

Direct comparisons of the 2 strategies to date have largely been single-institutional studies or multi-institutional analyses based on administrative databases.^3^^,^^4^^,^^7^^,^^8^^,^^12^^,^^15^, ^16^, ^17^ Despite attempts of propensity score matching, few studies could fully adjust for important patient-level risk factors, including prematurity, low birth weight, weight at surgery, extracardiac anomalies, hypoplastic pulmonary arteries, and pulmonary valve annulus,^3^^,^^7^^,^^18^^,^^19^ all of which were associated with adverse outcomes.^12^^,^^14^^,^^19^, ^20^, ^21^ Furthermore, some studies have combined patients with pulmonary stenosis and pulmonary atresia,^7^^,^^18^ the latter of which falls on the worst end of the spectrum of disease severity.^22^

At the Royal Children’s Hospital (RCH) in Melbourne, Australia, the institutional policy for the past 40 years has been to perform early systemic-to-pulmonary shunting for symptomatic infants before 4 months of age followed by transatrial-transpulmonary repair.^20^^,^^23^^,^^24^ In contrast, at the Children’s National Hospital (CNH), Washington, DC, the institutional policy since 2004 has been early PR for symptomatic young infants predominantly via the transventricular approach.^6^^,^^25^ To compare these 2 strategies, we performed a retrospective, propensity score–matched comparison of the outcomes at these institutions.

## Methods

### Patients

The SR group comprised all consecutive infants younger than 4 months of age who presented with symptomatic TOF and underwent palliative systemic-to-pulmonary shunt at the RCH between 1993 and 2021. The PR group comprised all consecutive infants younger than 4 months of age with symptomatic TOF who underwent PR at the CNH between 2004 and 2018. The indications for intervention included cyanosis, cyanotic spells, or ductal-dependent pulmonary flow. We have previously reported our perioperative management protocols and the sizes of shunts used.^20^^,^^25^ Patients with pulmonary atresia, absent pulmonary valve, or atrioventricular septal defect were excluded.

Neonates were aged 30 days or younger. Prematurity at birth was <37 weeks of gestation. Low birth weight was <2.5 kg. Nonelective procedures were performed within 2 days of the surgical decision. Respiratory insufficiency was defined as need for mechanical ventilation >120 hours or failed extubation. Reintervention was any unplanned surgical or transcatheter cardiothoracic intervention after the index procedure.

Medical records were reviewed for perioperative data. Follow-up of recent clinical status was obtained from correspondence with the patients’ cardiologists or general practitioners. The study design was approved by the respective ethics committees of the 2 institutions (RCH: #38341, April 5, 2019; CNH: #13608, February 13, 2020). The need for individual patient consent was waived because of the study’s retrospective design.

The index procedure was the first procedure performed in each treatment pathway, ie initial shunt palliation for the SR group, or single-stage repair for the PR group.

The primary outcome was mortality after the index procedure. Secondary outcomes included dichotomous end points (hospital complications and reinterventions) and continuous parameters (cardiopulmonary bypass [CPB] time, crossclamp time [XCT], postoperative right ventricular outflow tract [RVOT] peak gradient, duration of mechanical ventilation, intensive care unit [ICU] stay, and postoperative hospital stay). In the SR pathway, CR was considered an obligatory event and thus not a reintervention.

### Statistical Analysis

Statistical analysis was performed using Stata, version 14 (StataCorp). Categorical variables were expressed as frequency (percentage) and compared using the Fisher exact test. Normality of continuous variables were assessed with the Shapiro-Wilk test. Skewed continuous variables were expressed as median (interquartile range [IQR]) and compared using the Wilcoxon rank sum test. Analyses of dichotomous end points were performed using logistic regression. Time-dependent outcomes (death, reintervention) were analyzed using the Kaplan-Meier method and compared using the log-rank test. For analysis of reinterventions, patients exited the model in the event of death, but were retained in the model for a particular indication (RVOT obstruction, RV dilation, or pulmonary artery [PA] stenosis) if a reintervention occurred for another indication. Cox regression analysis was performed to examine the association of treatment strategy and baseline characteristics with time-related outcomes, specifically mortality and reintervention, with results reported as hazard ratio (HR) with 95% confidence interval (CI). Noncollinear variables with *P* < .10 were then entered into a multivariable model to determine independent predictors. Before performing Cox analyses, the proportional hazard assumption was verified by examining log-log survival plots. For time-dependent outcomes that failed to meet the proportional hazard assumption, Heaviside functions were used to estimate the effect of treatment during different phases of follow-up, and HRs were provided for each discrete intervals of time. The time intervals were determined from visual inspection of each survival curve, such that the difference between the estimated HR from each time interval is maximized. Schoenfeld residuals were then examined to verify the proportional hazard assumption for each interval.

Because baseline characteristics differed between the 2 groups, propensity score–matched analysis was performed to adjust for potential confounders. A propensity score was generated for each patient by performing a logistic regression in which treatment assignment (SR vs PR, with PR as the reference group) was regressed on baseline characteristics. The baseline characteristics used to generate the propensity score were those known to be risk factors for adverse outcomes or likely associated with treatment strategy selection, including prematurity, preintervention prostaglandin, mechanical ventilation, weight, nonelective procedure, pulmonary valve annulus size, and branch PA size. Furthermore, age was included *a priori* to ensure balance. Once generated, patients were matched 1:1 on their propensity score without replacement with a fixed caliper width set at 0.2 of the standard deviation of the logit of the propensity score (0.049).^26^ The degree of balance of the baseline characteristics in the two matched groups was assessed using standardized mean difference, where a difference ≤10% was considered to have achieved satisfactory balance. Between the matched groups, difference in binary outcomes were analyzed using conditional logistic regression, continuous outcomes were analyzed using the Wilcoxon signed rank test, and time-dependent outcomes were analyzed using an adjusted log-rank test stratified by quintiles of the propensity scores.^26^

## Results

### Study Cohort

The study cohort consisted of 265 patients, including 143 in the SR group and 122 in the PR group. All patients in the SR group underwent surgical systemic-to-pulmonary shunt, including right modified Blalock-Taussig shunt (n = 87), central shunt (n = 38), left modified Blalock-Taussig shunt (n = 15), and classic Blalock-Taussig shunt (n = 3). Baseline characteristics are summarized in Table 1. The SR group were younger, lower in weight, had smaller pulmonary valve annuli, and more commonly required prostaglandin, mechanical ventilation, and non-elective procedures. Prematurity was more prevalent in the PR group.

**Table 1:** Baseline characteristics of unmatched groups undergoing staged repair versus primary repair

Baseline characteristics	n	Staged repair (n = 143, 54%)	Primary repair (n = 122, 46%)	Standardized mean difference (%)	*P* value
Age, d	265	35 (15-63)	44 (21-77)	−30.6	**.01**
Neonates (≤30)		65 (46%)	38 (31%)	–	–
31-60		39 (27%)	37 (30%)	–	–
61-90		22 (15%)	21 (17%)	–	–
91-125		17 (12%)	26 (21%)	–	–
Weight, kg	252	3.4 (2.7-4.1)	4.6 (3.6-5.6)	−78.5	**<.001**
Operative weight <2.5 kg	252	20 (14%)	6 (5%)	–	**<.01**
Female	265	61 (43%)	41 (34%)	−18.6	.16
Prematurity (<37 wk)	265	27 (19%)	37 (30%)	−26.7	**.03**
Low birth weight (<2.5 kg)	265	45 (31%)	37 (30%)	2.5	.89
Genetic abnormality	265	27 (19%)	27 (22%)	6.0	.65
DiGeorge syndrome		5 (3%)	10 (8%)	20.1	.12
Gastrointestinal tract anomaly	265	13 (9%)	10 (8%)	−3.2	.83
Respiratory tract anomaly	265	7 (5%)	6 (5%)	0.1	1.00
Prostaglandin infusion before index procedure	265	51 (36%)	13 (11%)	61.8	**<.01**
ECMO before index procedure	265	1 (0.7%)	1 (0.8%)	1.4	1.00
Mechanical ventilation before index procedure	265	34 (24%)	11 (9%)	40.5	**<.01**
Nonelective procedure	265	77 (54%)	39 (32%)	45.2	**<.001**
Pulmonary valve annulus diameter, mm	225	4.6 (4.0-5.5)	5.4 (4.1-6.5)	−52.1	**<.001**
MPA diameter, mm	91	4.0 (3.5-4.6)	4.7 (3.7-6.0)	58.1	.06
RPA diameter, mm	248	3.9 (3.3-4.4)	3.8 (3.2-4.5)	−4.9	.95
LPA diameter, mm	241	3.6 (3.0-4.0)	3.6 (2.9-4.7)	−13.8	.46

Values are in n (%) or median (interquartile range), unless otherwise specified. Bolded *P* values are statistically significant. *ECMO*, Extracorporeal membrane oxygenation; *MPA*, main pulmonary artery; *RPA*, right pulmonary artery; *LPA*, left pulmonary artery.

### Early Outcomes of Unmatched Groups

**Figure E1:**
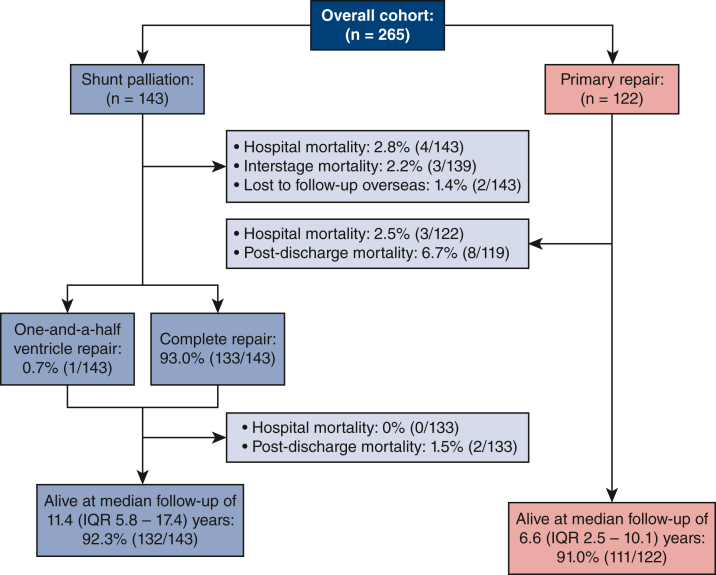
Cohort flow diagram depicting patient disposition in the staged repair and primary repair groups. *IQR*, Interquartile range.

Outcomes of unmatched groups are summarized in Table 2 and Figure E1. Hospital mortality after the index procedure was similar between the 2 groups (shunt: 2.8% [4/143] vs PR, 2.5% [3/122]; *P* = .86). In the SR group, interstage mortality was 2.2% (3/139), and 133 patients underwent staged CR with no hospital mortality. One patient underwent right modified Blalock-Taussig shunt at 6 days of age and was subsequently deemed not suitable for CR at 7 months of age, as her tricuspid valve was found intraoperatively to be significantly smaller than expected. She instead underwent bidirectional cavopulmonary connection and remained alive 13 years later.

**Table 2:** Outcomes of unmatched groups undergoing SR versus PR

Outcomes	SR (n = 143, 54%)					PR (n = 122, 46%)	
	First-stage shunt (n = 143)	*P* value shunt vs PR	Staged CR (n = 133)	*P* value staged CR vs PR	Cumulative SR (shunt + staged CR)∗ (n = 143)	PR	*P* value cumulative SR vs PR
Hospital mortality	4 (2.8%)	.86	0 (0%)	–	4 (2.8%)	3 (2.5%)	.86
Hospital complications							
Cardiac arrest	10 (7.0%)	.06	3 (2.3%)	.72	9 (6.3%)	2 (1.6%)	.08
Arrhythmia	6 (4.2%)	**<.01**	19 (14.3%)	.77	25 (17.5%)	19 (15.6%)	.68
Delayed chest closure	17 (11.9%)	.09	2 (1.5%)	.09	19 (13.3%)	7 (5.7%)	**.045**
ECMO	7 (4.9%)	.31	1 (0.8%)	.31	8 (5.6%)	3 (2.5%)	.22
Respiratory insufficiency†	32 (22.4%)	.39	10 (7.5%)	**<.001**	38 (26.6%)	30 (27.0%)	.71
Unplanned reintervention	29 (20.3%)	**<.001**	6 (4.5%)	.61	34 (23.8%)	4 (3.3%)	**<.001**
NEC requiring abdominal surgery	4 (2.8%)	–	0 (0%)	–	4 (2.8%)	0 (0%)	–
Other outcomes							
CPB, min	0 (0-56)	**<.001**	148 (111-179)	**<.001**	175 (138-212)	100 (81-118)	**<.001**
XCT, min	0 (0-0)	**<.001**	84 (66-106)	**<.001**	84 (66-106)	55 (44-66)	**<.001**
Mechanical ventilation, h	60 (37-137)	.34	19 (14-37)	**<.001**	97 (57-196)	53 (28-124)	**<.001**
ICU stay, d	4 (3-8)	**.01**	2 (1-3)	**<.001**	7 (4-12)	5 (3-11)	.08
Postoperative hospital stay, d	10 (7-17)	.89	7 (5-11)	**<.001**	18 (14-26)	10 (6-23)	**<.001**
Interstage duration, d	–	–	–	–	279 (175-376)	–	–
Details of complete repair							
Age, d	–	–	312 (205-441)	**<.001**	–	44 (21-77)	–
Weight, kg	–	–	7.4 (6.0-8.9)	**<.001**	–	4.6 (3.6-5.6)	–
MPA diameter, mm	–	–	6.3 (5.0-7.9)	**<.001**	–	4.7 (3.7-6.0)	–
RPA diameter, mm	–	–	6.0 (5.0-7.5)	**<.001**	–	3.8 (3.2-4.5)	–
LPA diameter, mm	–	–	6.0 (4.7-8.0)	**<.001**	–	3.6 (2.9-4.7)	–
Approach							
Transatrial	–	–	133 (100%)	**<.001**	-	7 (5.7%)	–
Transventricular	–	–	0 (0%)	–	–	113 (92.6%)	–
Combined	–	–	0 (0%)	–	–	2 (1.6%)	–
Patch location				**<.001**			
No patch	–	–	34 (25.6%)	–	–	8 (6.6%)	
Transannular	–	–	99 (74.4%)	–	–	50 (41.0%)	–
RV only	–	–	0 (0%)	–	–	56 (45.9%)	
RV and PA	–	–	0 (0%)	–	–	9 (7.4%)	
Valve-sparing	–	–	31 (23.3%)	**<.001**	–	71 (58.2%)	–
Pulmonary arterioplasty	–	–	45 (33.8%)	**.03**	–	58 (47.5%)	–
Postoperative RVOT peak gradient, mm Hg	–	–	24 (13-36)	**.04**	–	21 (10-30)	–
Follow-up, y	11.4 (5.8-17.4)	**<.001**	11.0 (5.7-16.9)	**<.001**	–	6.6 (2.5-10.1)	–
Postdischarge mortality	3 (2.2%)‡	.57	2 (1.5%)	.21	–	8 (6.7%)	–
2-y mortality§	8 (6.1%)	.68	0 (0%)	n/a	–	5 (4.9%)	–
10-y survival	95% (90%-98%)	.65	100%	**<.01**	–	90% (81%-95%)	–
10-y freedom from reintervention||	60% (50%-68%)	.70	68% (59%-76%)	.44	–	62% (52%-71%)	–
10-y freedom from reintervention after definitive repair							
RVOTO	–	–	80% (71%-86%)	.51	–	83% (74%-89%)	–
RV dilation	–	–	93% (86%-96%)	.14	–	98% (91%-99%)	–
PA stenosis	–	–	82% (74%-88%)	.85	–	81% (71%-88%)	–
PVR	–	–	93% (86%-96%)	.34	–	96% (89%-98%)	–
Reintervention-free survival at 10 y	57% (48%-65%)	.84	68% (59%-76%)	.10	–	58% (47%-68%)	–

Values are in n (%), median (interquartile range), or hazard ratio (95% confidence interval). Bolded *P* values are statistically significant. *SR*, Staged repair; *PR*, primary repair; *CR*, complete repair; *ECMO*, extracorporeal membrane oxygenation; *NEC*, necrotizing enterocolitis; *CPB*, cardiopulmonary bypass time; *XCT*, crossclamp time; *ICU*, intensive care unit; *MPA*, main pulmonary artery; *RPA*, right pulmonary artery; *LPA*, left pulmonary artery; *RV*, right ventricle; *PA*, pulmonary artery; *RVOT*, right ventricular outflow tract; *n/a*, not applicable; *RVOTO*, right ventricular outflow tract obstruction; *PVR*, pulmonary valve replacement.

∗ Cumulative number of hospital complications for the SR pathway may not be equal to the sum of shunt and CR, as some patients had the same complications after both procedures and were not counted twice.

† Respiratory insufficiency was defined as mechanical ventilation >120 hours or failed extubation. Data available for 143 patients in SR group and 111 in PR group.

‡ Postdischarge mortality for the shunt group occurred in the interstage period before postshunt repair.

§ Two-year mortality was calculated among 131 patients after shunt, 120 patients after staged CR, and 103 patients after PR, excluding survivors with <2 years of follow-up.

|| Freedom from reintervention after each procedure, with those after CR excluding interstage reinterventions after shunt.

In terms of secondary outcomes after the index procedure, the SR group had shorter durations of CPB, XCT, and ICU stay and similar durations of mechanical ventilation and postoperative hospital stay. The prevalence of cardiac arrest, delayed chest closure, need for extracorporeal membrane oxygenation and respiratory insufficiency were similar, whilst the prevalence of arrhythmia was lower in the SR group. However, the SR group more commonly required unplanned reinterventions before discharge, with 7.0% (10/143) requiring a shunt revision. The indications for reinterventions are summarized in Table E1. Four (2.8%, 4/143) patients had necrotizing enterocolitis after shunting and required abdominal surgery for bowel resection.

In terms of secondary outcomes after CR, the SR group had longer intraoperative CPB and XCT but shorter durations of postoperative mechanical ventilation, ICU stay, and hospital stay. The prevalence of respiratory insufficiency was greater in the PR group.

Cumulatively, the SR group had similar hospital mortality to the PR group but significantly greater prevalence of delayed chest closure and unplanned in-hospital reinterventions. Furthermore, the cumulative durations of CPB, XCT, mechanical ventilation, and postoperative hospital stay were shorter in the PR group.

### CR in Unmatched Groups

Details of CR in the unmatched groups are summarized in Table 2. Patients in the SR group were older, heavier, and had significantly larger branch PA size. All patients in the SR group underwent transatrial repair, whereas the majority in the PR group underwent transventricular repair. In the SR group, 74.4% (99/133) had a limited transannular patch (TAP),^20^ whereas 25.6% (34/133) required no patch in the RVOT. In the PR group, 41.0% (50/122) required a TAP, 45.9% (56/122) required an infundibular patch, 7.4% (9/122) required an infundibular and a main PA patch, and only 6.6% (8/122) required no patch. Valve-sparing repair and concomitant PA arterioplasty were more commonly performed in the PR group. Postoperative RVOT peak gradient on discharge was greater in the SR group.

### Late Outcomes of Unmatched Groups

**Figure 1:**
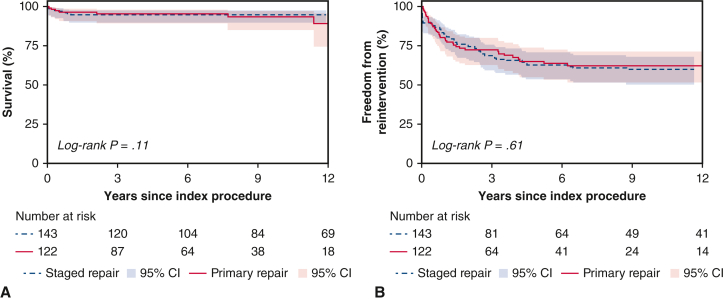
Unmatched comparison of (A) survival and (B) freedom from reintervention after index procedure, stratified according to treatment strategy. *CI*, Confidence interval.

The unmatched cohort was followed for median 8.3 years (IQR, 8.1-13.4 years) after index procedure. Kaplan-Meier survival (SR, 95%; 95% CI, 90%-98% vs PR, 90%; 95% CI, 81%-95%; *P* = .65; Figure 1, *A*) and freedom from reintervention (SR, 60%; 95% CI, 50%-68% vs PR, 62%; 95% CI, 52%-71%; *P* = .70; Figure 1, *B*) at 10 years were similar between the 2 groups. With SR as the reference group, there was no difference in the long-term hazard of reintervention (HR 0.92; 95% CI, 0.61-1.39, *P* = .70). However, early reintervention hazard (<6 months after index procedure) was lower in the PR group (HR, 0.22; 95% CI, 0.11-0.48, *P* < .001). The indications for reinterventions are summarized in Table E1. Almost one quarter (22.1% [27/119]) of PR survivors required percutaneous balloon valvuloplasty or PA angioplasty. Reintervention-free survival at 10 years was 57% (95% CI, 48%-65%) for the SR group and 58% (95% CI, 47%-68%) for the PR group (*P* = .84).

Predictors of mortality and reintervention after the index procedure are shown in Table E2. On univariable analysis, prematurity, low birth weight, and preoperative mechanical ventilation were associated with increased risk of mortality, whereas larger left PA size was associated with lower risk. Younger age was associated with greater risk of reinterventions. On multivariable analysis, prematurity was an independent predictor of mortality, whilst younger age was an independent predictor of reinterventions.

**Figure 2:**
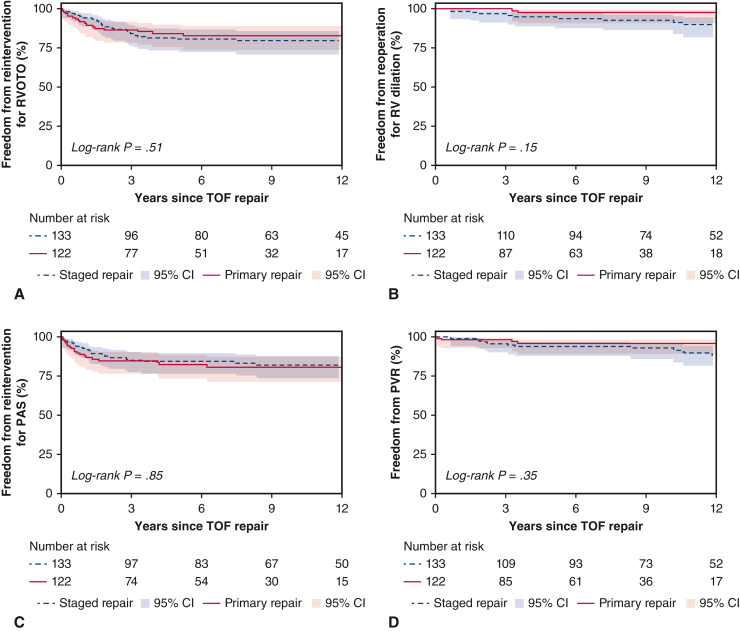
Unmatched comparison of freedom from reintervention for (A) right ventricular outflow tract obstruction (*RVOTO*); (B) right ventricular (*RV*) dilation; (C) pulmonary artery stenosis (PAS); and (D) pulmonary valve replacement (*PVR*) after complete repair, stratified according to treatment strategy. *TOF*, Tetralogy of Fallot; *CI*, confidence interval.

Freedom from reinterventions after CR for each indication is presented in Figure 2, with no significant difference between the 2 groups.

### Early Outcomes of Matched Groups

Propensity-score matching yielded 57 well balanced pairs (Table 3 and Figure E3). Outcomes of the matched groups are summarized in Table 4. Hospital mortality after the index procedure was similar (shunt, 1.8% [1/57] vs PR, 3.5% [2/57]; *P* = .57). There was no interstage mortality and no hospital mortality after CR in the SR group.

**Table 3:** Baseline characteristics of propensity score–matched groups undergoing staged repair versus primary repair

Baseline characteristics	n	Staged repair (n = 57)	Primary repair (n = 57)	Standardized mean difference (%)	*P* value
Age, d	114	52 (21-73)	42 (15-84)	−5.5	
Neonates (≤30)		19 (33%)	20 (35%)	–	.57
31-60		14 (25%)	16 (28%)	–	–
61-90		15 (26%)	9 (16%)	–	–
91-125		9 (16%)	12 (21%)	–	–
Weight, kg	114	3.7 (3.1-4.4)	3.9 (3.1-4.8)	−10.6	
Operative weight <2.5 kg		6 (11%)	6 (11%)	–	1.00
Female	114	20 (35%)	20 (35%)	0.0	1.00
Prematurity (<37 wk)	114	9 (16%)	8 (14%)	−4.1	1.00
Low birth weight (<2.5 kg)	114	13 (23%)	10 (18%)	−11.3	.64
Genetic abnormality	114	14 (25%)	17 (30%)	11.8	.83
DiGeorge syndrome	114	3 (5%)	7 (12%)	29.9	.32
Gastrointestinal tract anomaly	114	3 (5%)	5 (9%)	12.4	.72
Respiratory tract anomaly	114	2 (4%)	3 (5%)	8.1	1.00
Prostaglandin infusion before index procedure	114	13 (23%)	12 (21%)	−4.3	1.00
ECMO before index procedure	114	0 (0%)	1 (2%)	20.1	1.00
Mechanical ventilation before index procedure	114	8 (14%)	7 (12%)	−4.8	1.00
Nonelective procedure	114	24 (42%)	27 (47%)	10.9	.71
Pulmonary valve annulus diameter, mm	114	5.0 (4.3-6.0)	5.0 (3.8-6.1)	−2.1	.81
MPA diameter, mm	47	4.0 (3.4-5.3)	4.4 (3.5-6.0)	21.1	.39
RPA diameter, mm	110	3.8 (3.3-4.2)	3.5 (2.9-4.4)	−5.2	.42
LPA diameter, mm	109	3.7 (3.2-4.1)	3.4 (2.8-4.6)	4.7	.65

Values are in n (%) or median (interquartile range), unless otherwise specified. *ECMO*, Extracorporeal membrane oxygenation; *MPA*, main pulmonary artery; *RPA*, right pulmonary artery; *LPA*, left pulmonary artery.

**Table 4:** Outcomes of propensity score–matched groups undergoing SR versus PR

Outcomes	SR (n = 57)					PR (n = 57)	
	First-stage shunt (n = 57)	*P* value shunt vs PR	Staged CR (n = 56)	*P* value staged CR vs PR	Cumulative SR (shunt + staged CR)∗ (n = 57)	PR	*P* value cumulative SR vs PR
Hospital mortality	1 (1.8%)	.57	0 (0%)	n/a	1 (1.8%)	2 (3.5%)	.57
Hospital complications							
Cardiac arrest	6 (10.5%)	.09	0 (0%)	n/a	6 (10.5%)	1 (1.8%)	.09
Arrhythmia	4 (7.0%)	.22	10 (17.9%)	.57	14 (24.6%)	8 (14.0%)	.12
Delayed chest closure	8 (14.0%)	.41	0 (0%)	n/a	8 (14.0%)	5 (8.8%)	.41
ECMO	5 (8.8%)	.22	0 (0%)	n/a	5 (8.8%)	2 (3.5%)	.22
Respiratory insufficiency†	15 (26.3%)	.66	4 (7.1%)	**.03**	16 (28.1%)	14 (24.6%)	.68
Unplanned reintervention	13 (22.8%)	**.02**	1 (1.8%)	.34	13 (22.8%)	3 (5.3%)	**.02**
NEC requiring abdominal surgery	3 (5.3%)	–	0 (0%)	–	3 (5.3%)	0 (0%)	–
Other outcomes							
CPB, min	47 (0-58)	**<.001**	144 (112-178)	**<.001**	174 (150-235)	101 (83-123)	**<.001**
XCT, min	0 (0-0)	**<.001**	84 (71-102)	**<.001**	84 (71-102)	56 (46-66)	**<.001**
Mechanical ventilation, h	58 (35-166)	.35	19 (13-27)	**<.01**	86 (47-203)	72 (26-148)	**.03**
ICU stay, d	4 (2-8)	.57	2 (1-3)	**<.001**	6 (4-13)	5 (3-11)	.37
Postoperative hospital stay, d	10 (6-16)	.24	6 (5-11)	**<.01**	18 (13-25)	10 (6-26)	.33
Interstage duration, d	–	–	–	–	270 (149-376)	–	–
Details of complete repair							
Age, d	–	–	314 (210-439)	**<.001**	–	42 (15-84)	–
Weight, kg	–	–	7.6 (6.7-9.0)	**<.001**	–	3.9 (3.1-4.8)	–
MPA diameter, mm	–	–	7.3 (6.2-8.0)	**<.001**	–	4.4 (3.5-6.0)	–
RPA diameter, mm	–	–	6.0 (5.0-7.5)	**<.001**	–	3.5 (2.9-4.4)	–
LPA diameter, mm	–	–	5.6 (4.2-7.4)	**<.001**	–	3.4 (2.8-4.6)	–
Approach				**<.001**			
Transatrial	–	–	56 (100%)	–	–	4 (7.0%)	–
Transventricular	–	–	0 (0%)	–	–	52 (92.2%)	–
Combined	–	–	0 (0%)	–	–	1 (1.8%)	–
Patch location				**<.001**			
No patch	–	–	16 (28.6%)	–	–	4 (7.1%)	–
Transannular	–	–	40 (71.4%)	–	–	22 (38.6%)	–
RV only	–	–	0 (0%)	–	–	26 (45.6%)	–
RV and PA	–	–	0 (0%)	–	–	5 (8.8%)	–
Valve-sparing	–	–	14 (25.0%)	**.001**	–	34 (59.6%)	–
Pulmonary arterioplasty	–	–	18 (32.1%)	.18	–	27 (47.3%)	–
Postoperative RVOT peak gradient, mm Hg	–	–	26 (11-35)	.62	–	24 (14-32)	–
Follow-up, y	9.8 (4.9-15.6)	**<.01**	9.0 (4.9-14.2)	**.02**	–	7.1 (3.1-9.6)	–
Postdischarge mortality‡	0 (0%)	n/a	1 (1.8%)	.14	–	5 (8.8%)	–
2-y mortality§	1 (2.1%)	.34	0 (0%)	n/a	–	3 (6.1%)	–
10-y survival	98% (88%-99.8%)	**.02**	100%	**<.01**	–	85% (69%-93%)	–
10-y freedom from reintervention||	55% (39%-68%)	.85	64% (48%-76%)	.47	–	59% (43%-71%)	–
10-y freedom from reintervention after definitive repair							
RVOTO	–	–	76% (61%-86%)	.80	–	80% (64%-89%)	–
RV dilation	–	–	93% (81%-98%)	.94	–	95% (82%-99%)	–
PA stenosis	–	–	74% (58%-84%)	.77	–	81% (67%-89%)	–
PVR	–	–	88% (72%-95%)	.95	–	91% (79%-97%)	–
Reintervention-free survival at 10 y	54% (38%-67%)	.85	63% (48%-76%)	.22	–	52% (37%-66%)	–

Values are in n (%), median (interquartile range), or hazard ratio (95% confidence interval). Bolded *P* values are statistically significant. *SR*, Staged repair; *PR*, primary repair; *CR*, complete repair; *n/a*, not applicable; *ECMO*, extracorporeal membrane oxygenation; *NEC*, necrotizing enterocolitis; *CPB*, cardiopulmonary bypass time; *XCT*, crossclamp time; *ICU*, intensive care unit; *MPA*, main pulmonary artery; *RPA*, right pulmonary artery; *LPA*, left pulmonary artery; *RV*, right ventricle; *PA*, pulmonary artery; *RVOT*, right ventricular outflow tract; *RVOTO*, right ventricular outflow tract obstruction; *PVR*, pulmonary valve replacement.

∗ Cumulative number of hospital complications for the SR pathway may not be equal to the sum of shunt and CR, as some patients had the same complications after both procedures and were not counted twice.

† Respiratory insufficiency was defined as mechanical ventilation >120 hours or failed extubation.

‡ Postdischarge mortality for the shunt group occurred in the interstage period before post-shunt repair.

§ Two-year mortality was calculated among 48 patients after shunt, 46 patients after staged CR, and 49 patients after PR, excluding survivors with <2 years of follow-up.

|| Freedom from reintervention after each procedure, with those after CR excluding interstage reinterventions after shunt.

In terms of secondary outcomes after the index procedure, the 2 groups had similar durations of mechanical ventilation, ICU stay, and hospital stay. The prevalence of cardiorespiratory complications was similar. Consistent with the unmatched comparison, the matched SR group required more unplanned reinterventions before discharge. Three (5.3%, 3/57) patients had necrotizing enterocolitis after shunting and required abdominal surgery for bowel resection.

In terms of secondary outcomes after CR, the SR group had shorter durations of mechanical ventilation, ICU stay, and postoperative hospital stay but longer durations of intraoperative CPB and XCT. Consistent with the unmatched comparison, respiratory insufficiency was more prevalent in the PR group.

Cumulatively, 2-staged SR resulted in similar hospital mortality as PR. The prevalence of cardiorespiratory complications was similar except for unplanned reinterventions, which were again more prevalent in the SR group. Consistent with the unmatched comparison, the cumulative durations of CPB, XCT, and mechanical ventilation were shorter in the PR group.

### CR in Matched Groups

Details of CR in the matched cohorts are summarized in Table 4. The SR group were younger, heavier, had significantly larger branch PA size, more commonly required a TAP, and less commonly had valve-sparing repair. However, in contrast to the unmatched comparison, the incidence of concomitant PA arterioplasty and the postoperative RVOT peak gradient were similar between the matched groups.

### Late Outcomes of Matched Groups

**Figure 3:**
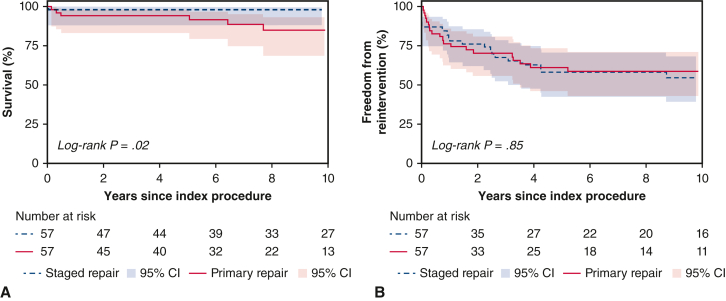
Propensity score–matched comparison of (A) survival and (B) freedom from reintervention after index procedure, stratified according to treatment strategy. *CI*, Confidence interval.

**Figure E2:**
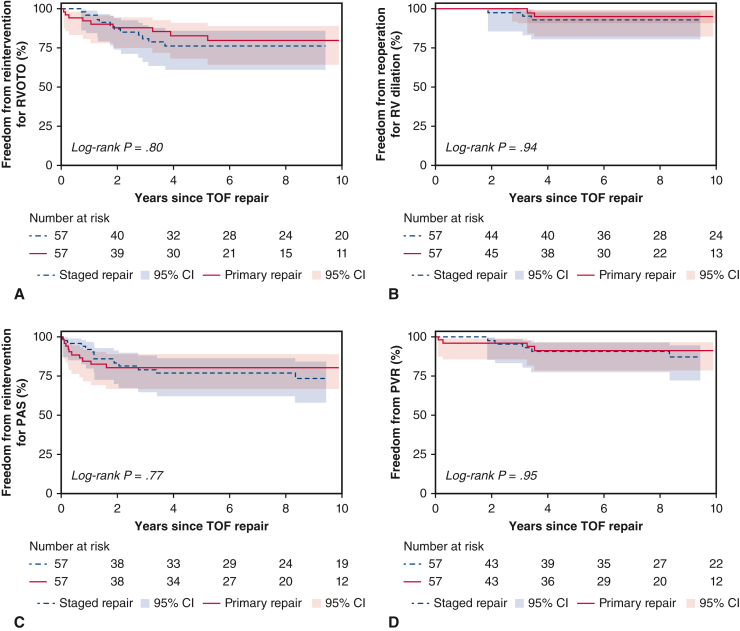
Propensity score-matched comparison of freedom from reintervention for (A) right ventricular outflow tract obstruction (*RVOTO*); (B) right ventricular (*RV*) dilation; (C) pulmonary artery stenosis (*PAS*); and (D) pulmonary valve replacement (*PVR*) after complete repair, stratified according to treatment strategy. 95% CI. *TOF*, Tetralogy of Fallot; *CI*, confidence interval.

**Figure E3:**
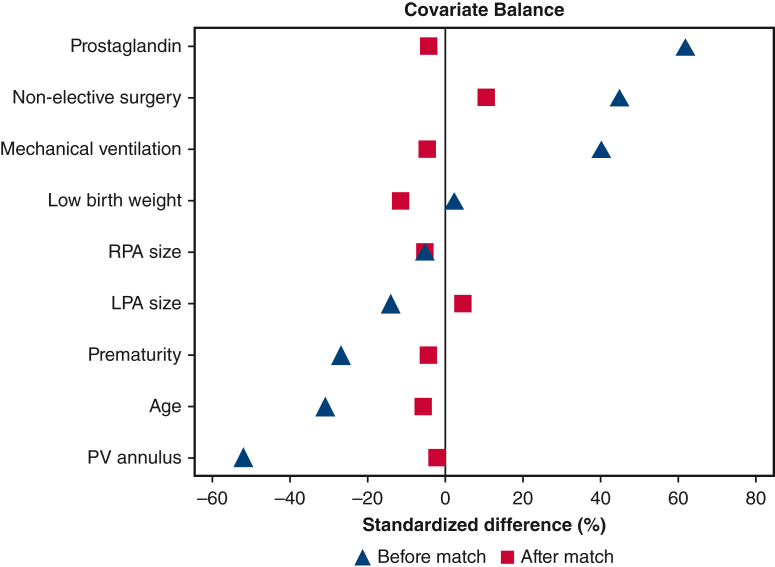
Covariate balance plot for determining adequacy of propensity score matching. Values on the horizontal axis represent the standardized difference between each group. *Triangles*: standardized difference before matching; *squares*: standardized difference after matching. *RPA*, Right pulmonary artery; *LPA*, left pulmonary artery; *PV*, pulmonary valve.

The matched cohort was followed for median 7.7 (IQR, 3.9-12.2) years after index procedure. Survival at 10 years was greater in the matched SR group (98%; 95% CI, 88%-99.8% vs 85%; 95% CI, 69%-93%; *P* = .02; Figure 3, *A*). In the SR group, the only early mortality occurred in a 102-day old infant born premature with low birth weight who had poor cardiac function and global cerebral ischemia after undergoing central shunt and was palliated 2 days later in view of poor prognosis. The only late mortality occurred in a patient with Goldenhar syndrome at 20 years after index procedure. In the PR group, early mortality occurred in a 4-month-old infant with Down syndrome because of myocardial infarction at 4 months after repair (age at repair: 19 days); and in a 2-month-old infant with chromosome 18q deletion syndrome as the result of sepsis at 53 days after repair (age at repair: 11 days). There were 5 late mortalities in the PR group: a 7-month-old infant, as the result of recurrent sepsis, who had mediastinitis and infective endocarditis after repair at 39 days of age and re-presented 6 months later with fever and gastrointestinal bleeding; a 5-year-old child, as the result of sepsis, who presented 5 years after repair with respiratory failure because of respiratory syncytial virus infection and renal impairment requiring dialysis; a 7-year-old child, as the result of sepsis, who presented 7 years after repair with respiratory failure and bowel ischemia; a 12-year-old child with Cornelia de Lange syndrome and chronic lung disease, as the result of respiratory failure at 12 years after repair; and a 6-year-old child with trisomy 10p who died of unknown cause at 6 years after repair. Freedom from reintervention at 10 years was similar (SR, 55%; 95% CI, 39%-68% vs PR, 59%; 95% CI, 43%-71%; *P* = .85; Figure 3, *B*). Reintervention-free survival at 10 years was 54% (95% CI, 38%-67%) for the SR group and 52% (95% CI, 37%-66%) for the PR group (*P* = .85). Freedom from reintervention after CR for each indication is presented in Figure E2, with no difference between the matched groups.

## Discussion

In this 2-institution propensity score–matched comparison of symptomatic infants with TOF younger than 4 months of age, SR was associated with greater risk of early reinterventions after initial shunting but equivalent risk of late reinterventions as PR. Matched comparison of secondary outcomes confirmed that the cumulative burden of perioperative support was greater in the SR pathway. However, the cumulative morbidity of major in-hospital cardiorespiratory complications was similar. Importantly, all-cause mortality was significantly greater in the PR group, which was driven by noncardiac-related late mortalities.

Comparison of outcomes after SR versus PR for symptomatic young infants with TOF have been the subject of multiple multi-institutional studies, some of which have arrived at divergent conclusions despite examining the same database.^3^^,^^7^^,^^18^ Ramakrishnan and colleagues^3^ examined the Pediatric Health Information System (PHIS) database and propensity score-matched 554 patients undergoing PR with 267 patients younger than 90 days of age undergoing systemic-to-pulmonary shunt. Hospital mortality and resource use were similar with either strategy. However, this study was limited to the first hospital stay and did not account for the cumulative burden of the staged approach nor late reinterventions. Furthermore, matching was not adjusted for prematurity, weight, branch PA and pulmonary valve annulus size, which could have influenced the choice of treatment strategy.^1^^,^^12^ Importantly, the unmatched comparison revealed that the different options were not offered equally to all patients, with patients offered shunting being younger, having a greater prevalence of prematurity, extracardiac anomalies, and more likely to be prostaglandin-dependent, consistent with our own findings. Steiner and colleagues^4^ also examined the PHIS database and found that the 2 strategies yielded similar operative mortality of 6%, despite the shunt group having had a greater proportion of genetic syndrome and coronary artery anomaly. They also found that whilst it shifted the morbidity burden out of the neonatal period, the SR approach resulted in greater cumulative morbidity and resource use. Savla and colleagues^7^ also examined the PHIS database and propensity score-matched 1331 symptomatic neonates undergoing SR with 1032 undergoing PR. They concluded that neonatal repair resulted in more cardiac complications and greater early and 2-year mortality. However, this study included patients with pulmonary atresia, who fell on the worst end of the disease spectrum and would likely have disadvantaged neonatal repair.^2^^,^^22^ Furthermore, as much as 42% of the study cohort had less than 2-years of follow-up, which limited the study’s statistical justification.

Considering the limitations of the abovementioned studies, we performed a propensity score–matched comparison of patients operated at 2 institutions that have exclusively adopted each strategy, in attempt to reduce selection bias at the institutional level.

### Mortality

Published evidence comparing the 2 strategies among symptomatic young infants have mostly focused on in-hospital or early mortality, with some finding greater mortality after early repair^7^^,^^17^ and others finding no difference.^3^^,^^4^^,^^8^^,^^14^^,^^21^ Goldstein and colleagues^18^ found an increased hazard of early mortality (<4 months) after PR, but overall mortality at 5 years was similar. In comparison, we found no difference in hospital mortality. However, we demonstrated a significant attrition of late mortalities in the matched PR group at up to 10 years after repair, which appeared to be attributable to noncardiac-related causes that occurred late after surgery. Despite propensity score matching, it is possible that unmeasured patient-related confounders, rather than the surgical strategy, have led to this persistently elevated mortality risk in our PR group. Yet, the fact that both strategies resulted in low operative mortality (2%-3%) and equivalent cardiac-related mortality is reassuring.

### Reinterventions

Our study confirms previous findings that SR may be associated with more reinterventions than PR, specifically in the interstage period.^8^^,^^18^ However, long-term hazard of reintervention was similar. Goldstein and colleagues^18^ similarly found a greater hazard of early (<3 months) but not late reintervention in their SR group. Of note, more than one third of our interstage reinterventions were shunt revisions, consistent with our previous findings and others’ that shunt stenosis and thrombosis cause substantial morbidity in the SR pathway.^18^^,^^20^ In contrast, most reinterventions after PR were percutaneous balloon valvuloplasty or PA angioplasty, the majority occurring within the first year, which suggests that adequate relief of the RVOT and/or hypoplastic PAs may be a particular challenge for early repair in smaller infants. It appears that similar patients were reoperated at the RCH, but had transcatheter interventions at the CNH, due to institutional preferences.

Importantly, we reported for the first time the prevalence of late reinterventions after CR according to indications, where we found no difference between the 2 strategies. We have previously reported that patients undergoing SR have high rates of reintervention, with 40% freedom from reintervention at 20 years after CR and neonates having similar outcomes as older infants.^20^ Furthermore, previous palliation was a risk factor for reoperations related to right ventricular (RV) dilation and RVOT obstruction.^23^ In contrast, data on late reintervention after early PR in young infants have so far been limited, with the literature pointing to 20% to 40% reintervention rates at just 5 years after neonatal repair.^22^^,^^27^^,^^28^ Our current study demonstrates that both strategies resulted in substantial reintervention rates, with approximately 40% reintervention at 10-years and the majority of which occurring in the first 5 years.

### Techniques of CR

Proponents of the SR strategy have proposed that initial shunting may reduce the need for a transannular incision by facilitating somatic growth before CR, which in turn results in more valve-sparing repairs and fewer reinterventions.^10^^,^^11^ It appears that we had found the opposite in our study, with our SR group having had more TAPs and fewer valve-sparing repairs even after matching for pulmonary valve annulus size. This may relate to the different surgical techniques adopted at our respective institutions. The RCH has adopted an RV infundibulum sparing strategy with transatrial-transpulmonary repair, followed by a small TAP if the annulus was moderately or severely hypoplastic.^20^^,^^29^ In contrast, the CNH has adopted a transventricular approach, with ventriculotomy extending across the annulus followed by a relatively larger TAP in case of hypoplastic annulus, or separate patches to the main PA and infundibulum if required.^6^^,^^25^ As such, although our PR group had relatively more valve-sparing repairs, they also required more patches in the RVOT overall, particularly to the infundibulum. Proponents of non-neonatal repair have argued that the infundibulum plays an essential role for RV function and should be spared as much as possible, which may be difficult to perform in a small heart and therefore repair should be delayed beyond the neonatal period,^9^^,^^29^^,^^30^ Furthermore, evidence is still limited that valve-sparing repairs are durable in preventing pulmonary insufficiency and RV dilation in the long term.^1^ In fact, aggressive valve-sparing approaches that require a ventriculotomy may result in similar degrees of RV dilation as a TAP.^31^ In our study, there was no difference in reintervention for RV dilation in the first decade. However, most reinterventions for RV dilation occur in the second and third decades after repair.^23^ Continued follow-up is necessary to capture any longer-term difference between these 2 strategies.

### Limitations

This study was limited by its retrospective nature and cohort size, whereby propensity score matching could not account for unmeasured confounders and statistical analyses might have been underpowered. We have attempted to adjust for clinical factors that were known predictors of adverse outcomes or likely to influence the choice of surgical strategy, but we were unable to account for institutional factors such as periprocedural management nor patient factors such as race or socioeconomic status, which may impact long-term outcomes. Furthermore, there might have been some variation in the selection of patients between Australia and the United States, and those differences may explain some of the discrepancies in late outcomes observed.

## Conclusions

In this propensity score–matched comparison of symptomatic young infants with TOF younger than 4 months of age operated at 2 institutions with exclusive treatment strategies, the SR strategy was associated with similar cardiac-related mortality and reinterventions as the PR strategy at medium-term follow-up. The PR strategy was associated with more valve-sparing repairs but also more patching of the RVOT. Both strategies may be reasonable at centers with demonstrated expertise.

## Conflict of Interest Statement

Dr Brizard has served on the advisory board of Admedus. All other authors reported no conflicts of interest.

The *Journal* policy requires editors and reviewers to disclose conflicts of interest and to decline handling or reviewing manuscripts for which they may have a conflict of interest. The editors and reviewers of this article have no conflicts of interest.

## Figures and Tables

**Table E1:** Reinterventions among unmatched groups undergoing SR versus PR

	SR		PR
	Interstage after first-stage shunt (n = 139∗)	After second-stage CR (n = 133∗)	After single-stage PR (n = 119∗)
Follow-up duration, y	0.8 (0.5-1.0)	11.0 (5.7-16.9)	6.6 (2.5-10.1)
Types of reinterventions			
Shunt revision	10 (7%)	0	0
ECMO cannulation	5 (4%)	0	0
Chest reopening for suspected shunt thrombosis	4 (3%)	0	0
RVOT resection	3 (2%)†	19 (14%)	6 (5%)
Chest reopening for hemodynamic instability	2 (1%)	0	0
Chest reopening for pneumothorax/hemothorax	2 (1%)	0	0
Diaphragm plication	2 (1%)	0	0
PA reconstruction	1 (0.7%)	8 (6%)	0
Percutaneous balloon valvuloplasty	0	1 (0.8%)	7 (6%)
Percutaneous PA angioplasty and/or stent	0	7 (5%)	20 (17%)
Pulmonary valve replacement	0	17 (13%)	6 (5%)
Residual VSD closure	0	1 (0.8%)	0

Values are expressed as median (interquartile range) or n (%). *SR*, Staged repair; *PR*, primary repair; *CR*, complete repair; *ECMO*, extracorporeal membrane oxygenation; *RVOT*, right ventricular outflow tract; *PA*, pulmonary artery; *VSD*, ventricular septal defect.

∗ Number of hospital survivors after each procedure.

† Interstage RVOT resection without complete repair.

**Table E2:** Predictors of mortality and reintervention after index procedure

Risk factors	Mortality				Reintervention			
	Univariable analysis		Multivariable analysis		Univariable analysis		Multivariable analysis	
	HR (95% CI)	*P* value	HR (95% CI)	*P* value	HR (95%CI)	*P* value	HR (95% CI)	*P* value
Primary repair (vs staged repair)	1.27 (0.45-3.57)	.65	–	–	0.90 (0.60-1.36)	.61	–	–
Age (d)	0.99 (0.98-1.01)	.35	–	–	0.99 (0.986-0.998)	**.01**	0.99 (0.986-0.998)	**.01**
Neonates (≤30 d)	1.59 (0.59-4.25)	.36	–	–	1.42 (0.96-2.11)	.08	∗	–
Prematurity (<37 wk)	3.38 (1.26-9.08)	**.02**	3.18 (1.15-8.77)	**.03**	0.76 (0.45-1.26)	.28	–	–
Low birth weight (<2.5 kg)	3.87 (1.40-10.71)	**<.01**	∗	–	0.73 (0.46-1.16)	.19	–	–
Genetic abnormality	2.39 (0.87-6.61)	.09	2.34 (0.84-6.55)	.11	0.61 (0.35-1.08)	.09	0.61 (0.34-1.07)	.08
Prostaglandin infusion before index procedure	1.80 (0.65-4.95)	.26	–	–	1.19 (0.76-1.86)	.44	–	–
Mechanical ventilation before index procedure	3.16 (1.15-8.74)	**.03**	∗	–	1.13 (0.65-1.97)	.65	–	–
Pulmonary valve annulus, mm	0.85 (0.57-1.28)	.44	–	–	0.86 (0.74-1.01)	.06	∗	–
RPA diameter, mm	0.78 (0.47-1.30)	.34	–	–	0.87 (0.72-1.06)	.16	–	–
LPA diameter, mm	0.59 (0.36-0.97)	**.04**	0.67 (0.42-1.07)	.09	0.85 (0.71-1.02)	.08	∗	–
Nonelective procedure	1.32 (0.50-3.53)	.58	–	–	1.24 (0.83-1.83)	.29	–	–

Bolded *P* values are statistically significant. *HR*, Hazard ratio; *CI*, confidence interval; *RPA*, right pulmonary artery; *LPA*, left pulmonary artery.

∗ Collinear variable not entered into multivariable analysis.
